# Supplementary material for: Optimization of Electron Transport Layer Inkjet Printing Towards Fully Solution-Processable OLEDs
Source: Materials (Basel). 2025 Jul 9;18(14):3231. doi: 10.3390/ma18143231 (PMC12300066; doi:10.3390/ma18143231)
Supplement: Supplementary file 1 [file materials-18-03231-s001.zip › materials-3722210-supplementary.pdf]

Optimization of electron transport layer inkjet printing towards fully solution processable OLEDs

Riccardo Manfredi [1†], Carmela Tania Prontera [1,2†], Fabrizio Mariano [1*], Marco Pugliese [1*], Antonio Maggiore [1], Alessandra Zizzari [1], Marco Cinquino [1], Iolena Tarantini [3], Giuseppe Gigli [1,3] and Vincenzo Maiorano [1]

[1] CNR-NANOTEC – Institute of Nanotechnology, c/o Campus Ecotekne, Via Monteroni, Lecce, 73100, Italy

[2] Laboratory of Hydrogen and New Energy Vectors (TERIN-DEC-H2V) ENEA - C.R. Brindisi S.S.7 Appia, km 713,700 - 72100 Brindisi, Italy

[3] Department of Mathematics and Physics, University of Salento, via Monteroni, Lecce, 73100, Italy

† These Authors contributed equally

* email: fabrizio.mariano@nanotec.cnr.it, marco.pugliese@nanotec.cnr.it

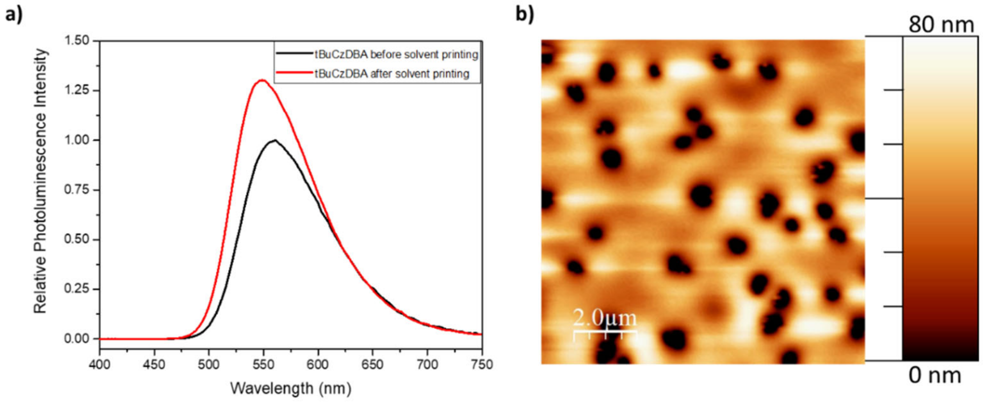

Figure S1: *a) PL spectrum of tBuCzDBA thin film before and after printing the solvent mixture #2 ; b) AFM image of printed TPBi, dissolved in the solvent mixture #2, on top of a tBuCzDBA film.*

Table S1: *Performance comparison of the prepared OLEDs*

Device	Maximum Luminance (cd/m^2)	Maximum Current Efficiency (cd/A)
**Evap. TPBi**	22340@333 mA/cm^2	24.1@2.8 mA/cm^2
**Spin. TPBi**	8728@267 mA/cm^2	7@31.7 mA/cm^2
**Print. TPBI 2 mg/mL**	4966@88 mA/cm^2	6.4@31.1 mA/cm^2
**Print. TPBi 2.5 mg/mL**	3713@51 mA/cm^2	11.1@6.3 mA/cm^2
**Print. TPBi 4 mg/mL**	5756@186 mA/cm^2	3.1@174.5 mA/cm^2
